# Remotely delivered weight management for people with long COVID and overweight: the randomized wait-list-controlled ReDIRECT trial

**DOI:** 10.1038/s41591-024-03384-x

**Published:** 2025-01-08

**Authors:** Emilie Combet, Laura Haag, Janice Richardson, Caroline E. Haig, Yvonne Cunningham, Heather L. Fraser, Naomi Brosnahan, Tracy Ibbotson, Jane Ormerod, Chris White, Emma McIntosh, Catherine A. O’Donnell, Naveed Sattar, Alex McConnachie, Michael E. J. Lean, David N. Blane

**Affiliations:** 1 Human Nutrition, School of Medicine, Dentistry and Nursing, University of Glasgow, Glasgow, UK; 2 School of Cardiovascular and Metabolic Health, University of Glasgow, Glasgow, UK; 3 Robertson Centre for Biostatistics, School of Health and Wellbeing, University of Glasgow, Glasgow, UK; 4 General Practice and Primary Care, School of Health and Wellbeing, University of Glasgow, Glasgow, UK; 5 Health Economics and Health Technology Assessment, School of Health and Wellbeing, University of Glasgow, Glasgow, UK; 6 Counterweight Ltd, London, UK; 7 Long Covid Scotland, Aberdeen, UK

**Keywords:** Viral infection, Obesity, Fatigue, Randomized controlled trials

## Abstract

Long COVID (LC) is a complex multisymptom condition with no known disease-modifying treatments. This wait-list-controlled open-label trial tested whether a remotely delivered structured weight management program could improve respective LC symptoms in people living with overweight. Adults with LC (symptoms >12 weeks) and body mass index >27 kg m^−2^ (>25 kg m^−2^ for South Asians) were randomized (*n* = 234, 1:1) to control (*n* = 116, usual care) or the remotely delivered structured weight management (*n* = 118, total diet replacement (850 kcal per day) for 12 weeks, followed by food reintroduction and weight loss maintenance support) via minimization and randomization (80:20) to balance dominant LC symptom, sex, age, ethnicity and postcode-based index of multiple deprivation between groups. The control group received the intervention after 6 months. Participants selected the dominant LC symptom they would most like to improve (fatigue, breathlessness, pain, anxiety/depression or other) as the prespecified respective primary outcome. Individual symptoms were assessed using validated questionnaires and a visual analog scale for those without prespecified scales. At 6 months, the primary outcome improved in the intervention group (change −1.16 (s.d. 1.42), *n* = 97 analyzed) compared with the control group (change −0.83 (s.d. 1.14), *n* = 117 analyzed) with a treatment effect of −0.34 (95% confidence interval −0.67 to −0.01), with no excess of serious adverse events. International Standard Randomised Controlled Trial Number Registry registration: ISRCTN12595520.

## Abstract (web summary)

In a randomized trial, a remotely delivered weight management intervention for people with long COVID and excess weight improved respective long COVID symptoms and quality of life and resulted in substantial weight loss when compared with a control group, with no serious adverse effects reported.

## Main

Long COVID (LC) is a multisystem condition that can affect approximately 10% of people following a coronavirus disease 2019 (COVID-19) infection^1,2^. These estimates are, however, highly variable, with the pooled prevalence of experiencing at least one symptom at follow-up of 34.82% (95% confidence interval (CI) 17.60–57.90) in people who contracted COVID-19 and were not hospitalized^3^. Although LC can affect anyone in the population, risk factors include female sex, socioeconomic disadvantage and raised body mass index (BMI)^4^. LC significantly affects quality of life and daily function, with common symptoms including fatigue, breathlessness and pain^5,6^. This is compounded by the profound impact of the condition on other aspects of life, such as employment and livelihoods, relationships with family and friends and participation in the community^1,7^. With over 200 symptoms reported in the context of LC, diagnosis and management are both challenging, which calls for inclusive approaches using respective primary outcomes to evaluate interventions^1,8^.

Excess weight is unlikely to play a central role in the etiology of LC given that people living with LC have a broad range of BMI and medical histories, but some symptoms of excess weight overlap and may aggravate those of LC. Furthermore, the metabolic and proinflammatory profile of excess body fat, the impact of excess weight on joints, blood vessels and lung function and the implications for future disease risk already associated with COVID-19 infections (for example, type 2 (T2) diabetes and cardiovascular diseases) provide a rationale for evaluating the impact of weight loss on LC among those also living with overweight and obesity^9^.

There is no broadly effective or disease-modifying treatment or cure for LC, with most trial research focusing on rehabilitation^10^. Specific treatments trialed, with varying levels of success, in subgroups of patients include pharmacological as well as non-pharmacological solutions such as pacing (balancing rest and activities in daily life) and supplementation^1^. Systemic inflammation and obesity have been identified as therapeutic targets for LC, but there is a gap in evidence-based strategies for people living with established LC and excess weight^9^.

Effective weight management has been shown to positively affect blood pressure, T2 diabetes, inflammation, lung function, pain, quality of life and fatigue^11–17^. Clinical benefits are observed with weight loss of 5–10%, yet more consistently with weight loss >10%, which usually demands input from a multidisciplinary team and professional dietary guidance^18–20^. The evidence so far has mostly involved in-person clinic attendance^12,21^; however, remote and digital programs may offer advantages in complex conditions where mobility and capacity to attend out-of-home appointments are limited.

The remotely delivered weight management for people living with LC and overweight trial (ReDIRECT) assessed whether remotely delivered evidence-based and professionally supported weight management could improve symptoms for people living with both LC and overweight. To do so, ReDIRECT implemented a respective patient-reported primary outcome, a continuous measure derived from the symptom score for the most important LC symptom selected by each participant at baseline (fatigue, breathlessness, pain, anxiety/depression or other).

## Results

### Patient disposition

**Fig. 1:**
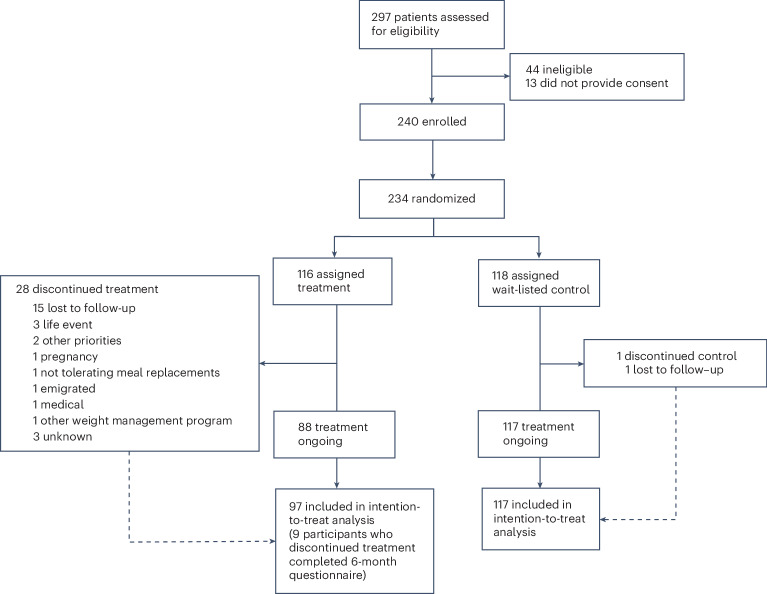
Participant flow through the ReDIRECT study. Trial profile showing the number of patients screened for eligibility, enrolled, randomized, allocated to treatment or control, and included in the intention-to-treat analysis at 6 months.

Applicants for inclusion (*n* = 297) were screened for enrollment, and *n* = 240 were recruited and provided consent between 23 December 2021 and 4 July 2022. Five did not complete the baseline assessment and were not randomized, and one withdrew consent after randomization, resulting in *n* = 234 randomized to the treatment (*n* = 116) and wait-listed control (*n* = 118) groups (Fig. 1).

Recruitment and baseline data have been published^22^; per-group baseline characteristics are presented in Table 1. Self-reported mean baseline weight was 101.9 kg (s.d. 20.4), ranging from 64 to 171.5 kg. A large proportion of participants had experienced weight gain following COVID infection (*n* = 105 (45%) in the intervention group, *n* = 110 (47%) in the control group), with approximately a third changing weight class status (National Institute for Health and Care Excellence) (*n* = 36 (31%) in the intervention group, *n* = 29 (25%) in the control group). There was no formal COVID testing for 56 participants for the first infection (a majority infected early in the pandemic), and for 32 participants when considering the first or second infection.

**Table 1:** Baseline characteristics

	Intervention group (*n* = 116)	Control group (*n* = 118)
**Sex,** ***n*** **(%)**		
Female	98 (84.5)	100 (84.7)
Male	17 (14.7)	17 (14.4)
Other	1 (0.9)	1 (0.8)
**Ethnicity**^**a**^, ***n*** **(%)**		
White	106 (91.4)	105 (89.0)
South Asian	5 (4.3)	5 (4.2)
Other Asian or Asian British	5 (4.3)	0 (0)
Black, African, Caribbean or Black British	0 (0)	2 (1.7)
Other or mixed ethnic group	0 (0)	6 (5.1)
Age (years)	46.4 (9.1)	46.1 (10.5)
**IMD quintile**, ***n*** **(%)**		
1 (most deprived)	18 (15.5)	13 (11.0)
2	22 (19.0)	22 (18.6)
3	16 (13.8)	29 (24.6)
4	23 (19.8)	28 (23.7)
5 (least deprived)	37 (31.9)	26 (22.0)
**Region**, ***n*** **(%)**		
England	76 (65.5)	73 (61.9)
Scotland	33 (28.4)	38 (32.2)
Wales	5 (4.3)	6 (5.1)
Northern Ireland	2 (1.7)	1 (0.8)
**Mean weight (kg)**	102.4 (21.5)	101.5 (19.4)
**Mean BMI (kg** **m**^**−**^^**2**^**)**	36.8 (7.5)	36.8 (6.5)
BMI white (*n* = 211, 90%)	36.9 (7.4)	37.0 (6.4)
BMI nonwhite^b^ (*n* = 23, 10%)	35.5 (8.4)	35.2 (7.2)
**Mean SBP (mmHg)**	130.7 (14.5)	132.2 (13.3)
**Mean DBP (mmHg)**	80.1 (9.1)	80.1 (8.8)
**Hypertension**, ***n*** **(%)**	12 (10.3)	20 (16.9)
**Known T2 diabetes mellitus**, ***n*** **(%)**	3 (2.6)	3 (2.5)
**Nominated LC symptom (primary outcome)**, ***n*** **(%)**		
Pain	14 (12.1)	14 (11.9)
Breathlessness	20 (17.2)	17 (14.4)
Fatigue	60 (51.7)	66 (55.9)
Anxiety/depression	1 (0.9)	2 (1.7)
Other	21 (18.1)	19 (16.1)
**Mean time since first (reported) COVID infection (months)**	16.8 (7.8)	16.8 (7.8)
**Timing of first COVID infection**, ***n*** **(%)**^**c**^		
Before 16 May 2021 (Alpha variant dominant)	82 (70.7)	88 (74.6)
Between 16 May 2021 and 19 December 2021 (Delta variant dominant)	22 (19.0)	15 (12.7)
Between 20 December 2021 and 1 March 2022 (Omicron BA.1 variant dominant)	10 (8.6)	13 (11.0)
After 1 March 2022 (Omicron BA.2 variant dominant)	2 (1.7)	2 (1.7)
**Number with confirmed diagnosis of COVID in first or second infection**, ***n*** **(%)**	100 (86)	102 (86)
**Vaccination status**, ***n*** **(%)**		
**At the time of contracting COVID first time**		
Not vaccinated	81 (69.8)	88 (74.6)
Vaccinated once	6 (5.2)	2 (1.7)
Vaccinated twice	18 (15.5)	10 (8.5)
Vaccinated thrice	11 (9.5)	15 (12.7)
**At recruitment**		
Not vaccinated	3 (2.6)	3 (2.5)
Vaccinated once	1 (0.9)	1 (0.8)
Vaccinated twice	9 (7.8)	10 (8.5)
Vaccinated thrice	103 (88.8)	104 (88.1)
**Mean time since becoming aware of LC (months)**	14.3 (7.3)	13.5 (7.4)
**Taking medications**^**d**^, ***n*** **(%)**		
0	23 (19.8%)	20 (16.9%)
1	16 (13.8%)	19 (16.1%)
2	15 (12.9%)	10 (8.5%)
3	17 (14.7%)	11 (9.3%)
4	12 (10.3%)	15 (12.7%)
5 or more	33 (28.4%)	43 (36.4%)

Data are *n* (%) or mean (s.d.), unless otherwise specified.

^a^There were no differences between groups at baseline, except for ethnicity, which is attributable to ethnic groups with fewer than *n* = 10.

^b^Ethnicities reported as nonwhite include South Asian, other Asians and Asian British.

^c^Dates for COVID-19 variance dominance are based on a report by the Office for National Statistics^37^.

^d^Medication includes prescribed and over the counter.

In the intervention arm, two (0.8%) participants withdrew at 3 months, and one (0.4%) withdrew at 6 months. The reasons for withdrawals were pregnancy, unknown reasons and emigration. In addition, nine (4%) participants were lost to follow-up at 3 months and 7 (3%) at 6 months. All but one participant lost to follow-up (at 6 months) were in the intervention arm. Data were available for the primary outcome at 3 months for 219 participants (94%, *n* = 101 in the intervention group, *n* = 118 in the delayed-entry control group) and at 6 months for 214 participants (91%, *n* = 97 intervention, *n* = 117 control).

Of the 116 people randomized to the weight management intervention group, most (109, 95%) started the program with total diet replacement (TDR), five chose an alternative dietary strategy (meal replacement (MR) *n* = 1, low-fat diet *n* = 1, low-carb diet *n* = 2, food-based plan not specified *n* = 1) and two did not start the program (planning pregnancy *n* = 1, loss to follow-up *n* = 1). Of those who started, *n* = 16 (14%) switched to an alternative during the first 3 months of the program (from TDR to MR *n* = 8, TDR to low carb *n* = 4, TDR to low fat *n* = 2, from MR to TDR *n* = 1, from low fat to MR *n* = 1) and *n* = 88 completed the weight management program at 6 months (76%).

The baseline characteristics of the *n* = 20 participants for whom a primary outcome measurement was unavailable at 6 months are presented in Supplementary Table 1, alongside descriptives from those for whom the outcome was collected. There were no statistically significant differences between groups, except for a greater proportion (65% versus 29%) of participants who did not complete the study at 6 months living in areas of higher socioeconomic deprivation (index of multiple deprivation (IMD) quintiles 1 and 2).

Up to 6 months, a median of 378 sachets (interquartile range 308–378) were issued to the 109 participants following TDR, with *n* = 77 (70.6%) issued the same or more sachets than stated in the protocol (378 sachets to complete TDR and food reintroduction stages).

### Primary outcome

The prespecified primary outcome was based on the participant-selected dominant LC symptom score. ‘Fatigue’ was the most frequently selected LC symptom that participants most wanted to improve (*n* = 126, 54%), followed by ‘breathlessness’ (*n* = 37, 16%), ‘pain’ (*n* = 28, 12%) and ‘anxiety/depression’ (*n* = 3, 1%). A further 40 (17%) selected an alternative ‘other’ symptom distinct from the four major symptoms listed previously. In total, 816 ‘other’ symptoms were reported, 432 in the intervention group and 384 in the control group.

The primary outcome for each participant was defined by an algorithm where a score of zero represented the average at baseline, with positive values indicating above-average severity, and an improvement in the patient-selected primary outcome being shown by negative changes over time (see Methods for more details). The study was designed to be able to detect a mean difference between groups at the primary analysis time point (6 months) of 0.5 points.

**Fig. 2:**
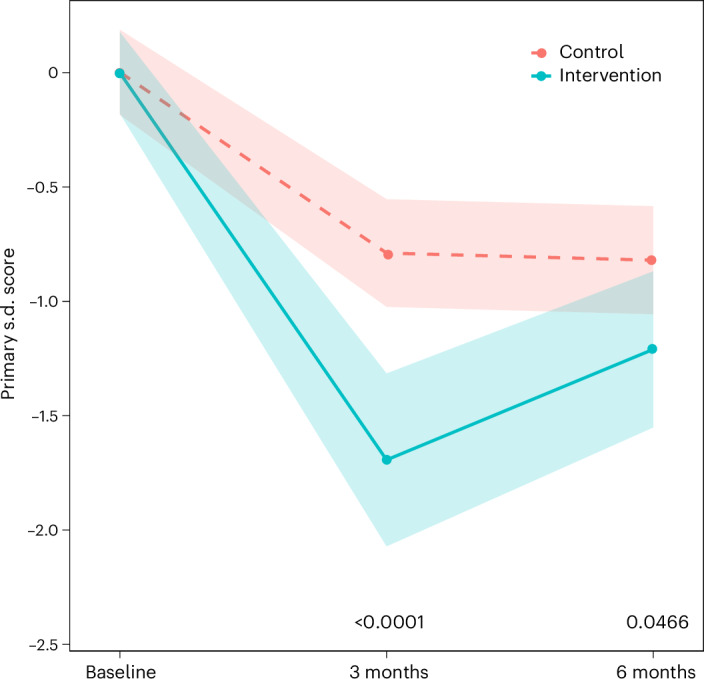
Change in the primary outcome (continuous measure derived from the symptom score for the most important LC symptom as selected by each participant at baseline (fatigue, breathlessness, pain, anxiety/depression or other)). The data are presented as means, with the shaded areas representing the 95% CIs. Two-sided *P* values (linear regression) represent the treatment effect at 3 months (*P* < 0.0001, *n* = 219) and 6 months (*P* = 0.0466, *n* = 214) compared with baseline. No adjustment is made for multiple comparisons.

At 6 months, the adjusted between-group mean difference favored the intervention (intervention effect estimate −0.34 (95% CI −0.67 to −0.01), *P* = 0.0466). This difference was more marked at 3 months (−0.90 (95% CI −1.27 to −0.53), *P* < 0.0001) (Fig. 2 and Supplementary Tables 2 and 3).

In *n* = 7 cases, ‘other’ symptoms selected as the primary outcome that matched a core symptom were recoded for sensitivity analysis (for example, ‘fatigued’ could be mapped onto the core symptom ‘fatigue’), with no change in the impact of the intervention at 6 months (−0.37 (95% CI −0.70 to −0.03), *P* = 0.0342) or 3 months (−0.94 (95% CI −1.31 to −0.56), *P* < 0.0001).

### Secondary outcomes

The four main symptoms (fatigue, pain, breathlessness and anxiety/depression) are reported as secondary outcomes for all participants, and ‘other’ for all participants who provided these data, regardless of whether these symptoms were identified as symptoms to improve and included in the primary outcome calculation.

**Fig. 3:**
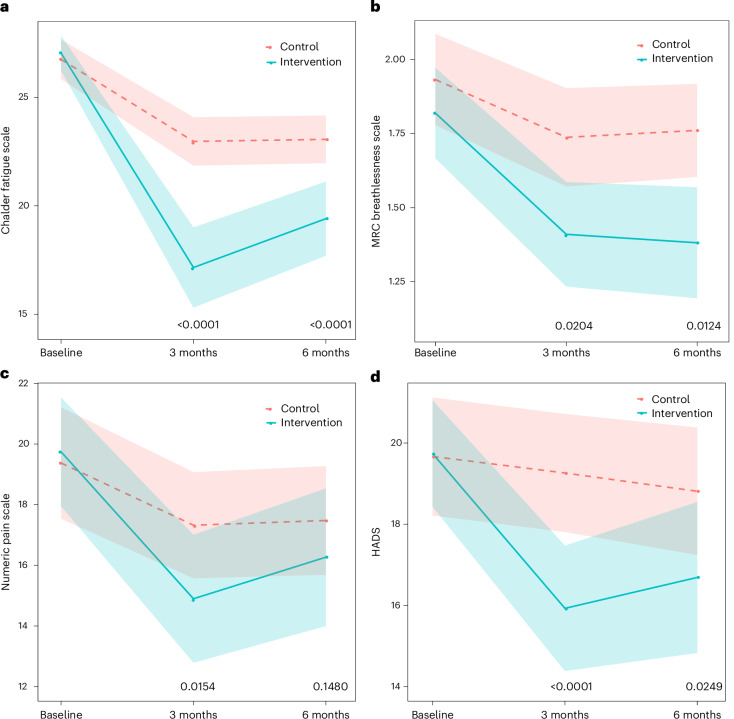
Change in LC symptoms as secondary outcomes. **a**–**d**, Fatigue (**a**), breathlessness (**b**), pain (**c**) and anxiety/depression (**d**). The data are presented as means, with the shaded areas representing the 95% CIs. Two-sided *P* values (linear regression) represent the treatment effect compared with baseline at 3 months for fatigue (*P* < 0.0001, *n* = 217), breathlessness (*P* = 0.0204, *n* = 218), pain (*P* = 0.0154, *n* = 217) and anxiety/depression (*P* < 0.0001, *n* = 217) and 6 months for fatigue (*P* < 0.0001, *n* = 214), breathlessness (*P* = 0.0124, *n* = 214), pain (*P* = 0.1480, *n* = 214) and anxiety/depression (*P* = 0.0249, *n* = 214). No adjustment is made for multiple comparisons.

**Fig. 4:**
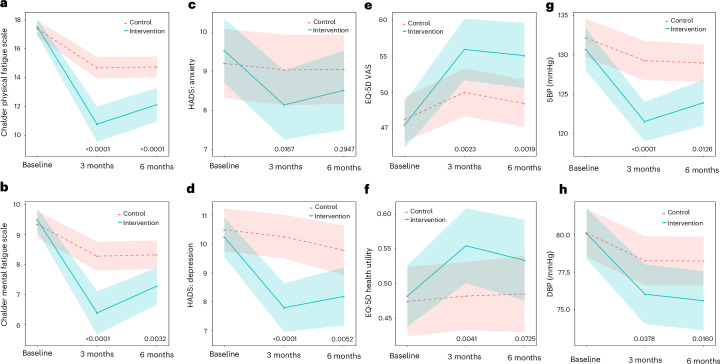
Change in LC symptoms as secondary outcomes at 6 months. **a**–**f**, Physical (**a**) and mental (**b**) fatigue; HADS anxiety (**c**) and depression (**d**); quality of life (EQ-5D visual scale (**e**) and utility (**f**)); systolic (**g**) and diastolic (**h**) blood pressure. The data are presented as means, with the shaded areas representing the 95% CIs. Two-sided *P* values (linear regression) represent the treatment effect compared with baseline: at 3 months for physical fatigue (*P* < 0.0001, *n* = 217), mental fatigue, (*P* < 0.0001, *n* = 217), HADS anxiety (*P* = 0.0167, *n* = 217), HADS depression, (*P* < 0.0001, *n* = 217), EQ-5D visual scale (*P* = 0.0023, *n* = 217), EQ-5D utility (*P* = 0.0041, *n* = 217), SBP (*P* < 0.0001, *n* = 216), DBP (*P* = 0.0378, *n* = 216) at 6 months for physical fatigue (*P* < 0.0001, *n* = 214), mental fatigue, (*P* = 0.0032, *n* = 214), HADS anxiety (*P* = 0.2947, *n* = 214), HADS depression, (*P* = 0.0052, *n* = 214), EQ-5D visual scale (*P* = 0.0019, *n* = 214), EQ-5D utility (*P* = 0.0725, *n* = 214), SBP (*P* = 0.0126, *n* = 212), DBP (*P* = 0.0160, *n* = 212). No adjustment is made for multiple comparisons.

At 6 months, the intervention improved all LC symptoms as secondary outcomes, except for pain (treatment effect −1.41 (95% CI −3.32 to 0.50), *P* = 0.1480) (Figs. 3 and 4).

Fatigue changed by −7.5 (s.d. 7.7) points in the intervention group and −3.7 (s.d. 5.5) points in the control at 6 months, with a treatment effect of −3.64 (95% CI −5.42 to −1.86), *P* < 0.0001 (Fig. 3a). Similar effects were seen for both the physical fatigue subscale (treatment effect −2.56 (95% CI −3.77 to −1.35), *P* < 0.0001) and the mental fatigue subscale (treatment effect −1.06 (95% CI −1.76 to −0.36), *P* = 0.0032) (Fig. 4a,b).

Breathlessness changed by −0.4 (s.d. 0.9) points in the intervention group and −0.2 (s.d. 0.8) points in the control, with a treatment effect of −0.27 (95% CI −0.48 to −0.06), *P* = 0.0124 (Fig. 3b).

Anxiety and depression changed by −2.9 (s.d. 7.2) points in the intervention group and −0.9 (s.d. 5.5) points in the control group, with a treatment effect of −1.94 (95% CI −3.64 to −0.25), *P* = 0.0249 (Fig. 3d). This was mostly attributable to the depression subscale (treatment effect −1.44 (95% CI −2.44 to −0.43), *P* = 0.0052), with no clear impact on the anxiety subscale (treatment effect −0.50 (95% CI −1.43 to 0.43), *P* = 0.2947) (Fig. 4c,d).

Other symptoms (rating on 10-point visual analog scale (VAS)), reported by *n* = 179 (76%), also changed by −2.0 (s.d. 2.9) points in the intervention group and −1.4 (s.d. 2.6) points in the control group, with a treatment effect of −0.82 (95% CI −1.46 to −0.19), *P* = 0.0113 (Supplementary Table 3).

Quality of life (EQ-5D VAS; Fig. 4e) changed by 10.9 (s.d. 18.2) points in the intervention group and 2.3 (s.d. 17.8) points in the control group, with a treatment effect of 7.55 (95% CI 2.82 to 12.29), *P* = 0.0019. While the between-group difference in the health utility score was not significant (treatment effect of 0.05 (95% CI −0.00 to 0.11), *P* = 0.0725) (Fig. 4f), when used to calculate quality-adjusted life years (QALYs), the QALY gain over time was significantly improved for the intervention group (*P* = 0.0017).

The number of hours of work missed per week due to health problems reduced by 3.38 (s.d. 13.97) in the intervention group and 2.34 (s.d. 14.03) in the control group, with a treatment effect of 2.44 (95% CI −0.43 to 5.31, *P* = 0.0949).

All improvements at 6 months were also observed at 3 months, with the addition of a significant treatment effect on pain and quality of life (health utility score) (Supplementary Table 2).

**Extended Data Fig. 1:**
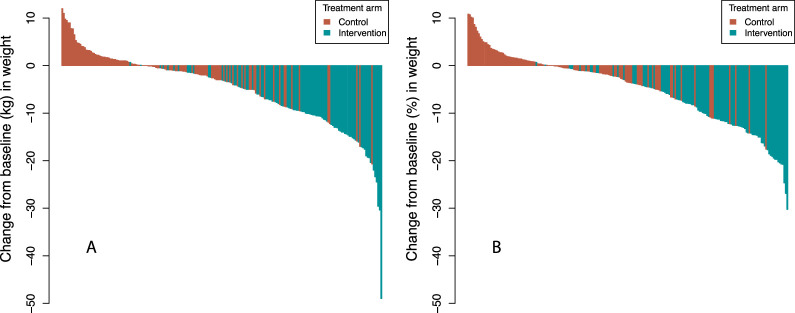
Change in weight at 6 months. Change from baseline in kg (**A**) or % of baseline weight (**B**).

Over 6 months, participants in the intervention group either maintained their baseline weight or lost weight (range of weight change −49.0 kg to +0.7 kg; Supplementary Tables 2 and 3 and Extended Data Fig. 1). In the control group, while the mean weight at 6 months was similar to baseline weight, some participants gained or lost large amounts (range −20.7 kg to +12.0 kg). Mean body weight in the intervention group fell by −9.8 kg (s.d. 6.0) after 3 months and by −10.3 kg (s.d. 7.5) at 6 months. Mean weight changes in the control group were small: −1.3 kg (s.d. 4.7) after 3 months and −0.7 kg (s.d. 5.2) after 6 months (adjusted group difference −9.79 kg (95% CI −11.5 to −8.1), *P* < 0.0001). These equate to a −10.3% change from the baseline (s.d. 6.4, range −30.2 to 0.7) for the intervention group and −0.6% (s.d. 4.9, range −17.6 to 10.8) for the control group.

Mean systolic blood pressure (SBP) (Fig. 4g) improved at 6 months, with a −6.6 (s.d. 14.1) mmHg reduction in the intervention group and −3.2 (s.d. 12.9) mmHg reduction in the control group (adjusted group difference −4.16 mmHg (95% CI −7.42 to −0.90), *P* = 0.0126). Diastolic blood pressure (DBP) (Fig. 4h) decreased in the intervention group at 6 months, with a −4.7 (s.d. 9.0) mmHg reduction and −1.8 (s.d. 9.1) mmHg reduction in the control group (adjusted group difference −2.73 mmHg (95% CI −4.95 to −0.51), *P* = 0.0160).

### Safety

A total of 16 serious adverse events (SAEs) were reported during the 6 months of the study, 10 in the control group and 6 in the intervention group (Table 2). There were no deaths. SAEs were similarly distributed between the first and second 3 months of the study in the control group (50% of events in days 0–90) and the intervention group (60% during days 0–90). SAEs were rated as severe (*n* = 6, 60%), moderate (*n* = 3, 30%) or mild (*n* = 1, 10%) for the control group and moderate (*n* = 2, 33%) or mild (*n* = 3, 50%) for the intervention group (one unclassified). Two SAEs (gallstone events) were considered related to the intervention but were not unexpected.

**Table 2:** SAEs (*n* = 16)

	All (*n* = 234)	Intervention (*n* = 116)	Control (*n* = 118)
Total number of SAEs	16	6	10
Number of participants with any SAE	15 (6.4%)	6 (5.2%)	9 (7.6%)
Gastrointestinal disorders	1 (<1%)	1 (<1%)	0
Hepatobiliary disorders	3 (1.3%)	2 (1.7%)	1 (<1%)
Infections and infestations	1 (<1%)	1 (<1%)	0
Injury, poisoning and procedural complications	1 (<1%)	0	1 (<1%)
Musculoskeletal and connective tissue disorders	1 (<1%)	0	1 (<1%)
Nervous system disorders	4 (1.7%)	0	4 (1.7%)
Pregnancy	1 (<1%)	1 (<1%)	0
Surgical and medical procedures	4 (1.7%)	2 (1.7%)	2 (1.7%)

### Exploratory outcomes

**Extended Data Fig. 2:**
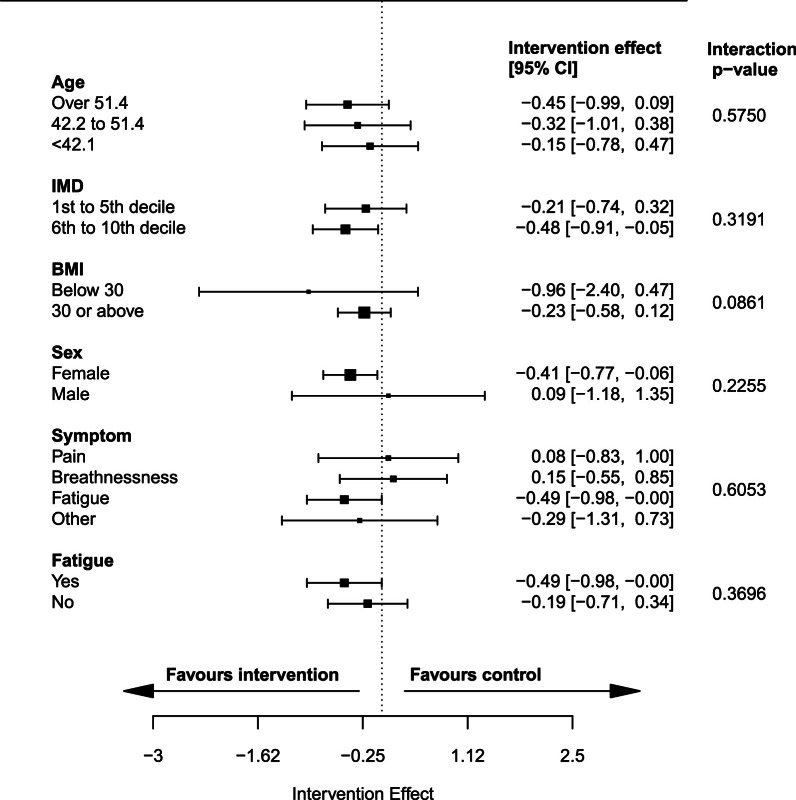
Difference in treatment effect based on age, deprivation level (IMD), baseline body mass index (BMI), sex, primary symptom selected (and whether this included fatigue or not). Data are presented as means with 95% confidence intervals. Two-sided p-values represent the interaction between subgroup and treatment (linear regression/ANCOVA), exploring age ( < 42.1: n = 71, 42.2 to 51.4: n = 69, >51.4: n = 74, p = 0.5750), IMD (1st to 5th decile: n = 86, 6th to 10th decile: n = 128, p = 0.3191), BMI (Below 30: n = 31, 30 or above: n = 183, p = 0.0861), sex (female: n = 182, male: n = 31, p = 0.2255), primary symptom selected (Pain: n = 26, Breathlessness: n = 35, Fatigue: n = 115, Other: n = 37, p = 0.6053), and fatigue as a primary symptom (Yes: n = 115, No: n = 99, p = 0.3696).

There was no difference in treatment effect for the primary outcome based on age, deprivation, BMI at baseline, sex or main symptom selected (Extended Data Fig. 2).

### Sensitivity analyses

All primary and secondary analyses were repeated with multiple imputations using chained equations to account for missing data, with similar results (Supplementary Table 4). Primary and secondary outcomes weighted by the inverse probability of follow-up are also presented in Supplementary Table 4.

Using only data from people with a positive test for their first infection (*n* = 178), the treatment effect on the primary outcome at 6 months was −0.379 (95% CI −0.769 to 0.011), *P* = 0.0565. This is similar to when data from people who had a positive test for their second infection were included (total *n* = 202 with a positive test for first or second infection) (−0.310 (95% CI −0.678 to 0.058), *P* = 0.0980).

### Post-hoc analyses

**Extended Data Fig. 3:**
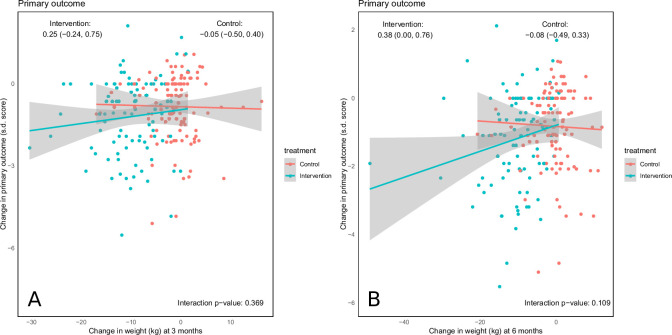
Crude interaction analysis between change in weight and effect of the intervention on the primary outcome. Data are presented as means with shaded areas representing 95% confidence intervals at 3 months with n = 216 (**A**) and 6 months with n = 212 (**B**). Estimates and p-values (two-tailed, linear regression) are given per 10 kg change in weight change, and no adjustments are made for multiple comparisons.

We explored the possible mediating role of weight loss as a key intervention component in primary outcome improvement, a post-hoc analysis not prespecified in our statistical analysis plan but suggested during the review process. There was no evidence of an interaction between weight loss at 3 or 6 months and the intervention effect on the primary outcome (Extended Data Fig. 3).

## Discussion

We found that a remotely delivered weight management intervention in people with LC and excess body weight effectively reduced LC symptoms that matter most to participants, alongside substantial weight loss. There was a significant treatment effect on the respective primary outcome, the LC symptom that each participant nominated as the one they most wanted to see improve. In our study, all individually self-reported LC symptoms (except pain) improved at 6 months in the intervention group compared with the control group, with a parallel improvement in quality of life. The intervention was safe, with no difference in reported SAEs.

The mean weight loss of 10 kg in the intervention group (1 kg in the control group) corresponded to meaningful reductions in blood pressure, notable considering that SBP has been identified as the leading contributor to the population-attributable fraction of all cardiovascular disease risk factors^23^. Given measured declines in blood pressure and known benefits of weight loss in reducing the risks for multiple other complications, the substantial intentional weight loss, if maintained, could have multiple other benefits for participants whose baseline BMI averaged around 35 kg m^−2^. As it is usual for weight management trials to require a stable baseline weight, it is worth noting that most study participants were not weight stable when they entered the trial, with >90% reporting weight gain following COVID infection, and National Institute for Health and Care Excellence weight class status changing for approximately a third of the participants^22^. This may have blunted the effect of the intervention.

We recognize several limitations but also strengths. The trial was conducted in real-life settings with few exclusions, but as with most studies of LC, the trial relied on patient-generated data, including self-reported weight. LC is a complex condition defined by lasting symptoms attributed to a COVID-19 infection. There are no objective diagnostic tests for LC. Although we recorded the self-reported timing of the first infection, we do not have specific information about the strain that people were exposed to, which limits any further sensitivity analysis linked to viral strain exposure^22^. The initial COVID infection was indicated by positive immunological tests, self-tested in most cases, which was often impossible in the early stages of the pandemic before the establishment of community testing. Indeed, in some cases, there was no formal testing for the first or second infection, so inclusion was based on a reported history of a COVID-like illness during the pandemic and subsequent persistent symptoms. Sensitivity analysis focusing only on people with a positive test shows that the treatment effect is maintained, despite losing power.

The improvements in symptoms with the intervention were consistent at 3 and 6 months with some decline in the treatment effect between 3 and 6 months despite well-maintained weight loss. Follow-up analysis at 12 months will indicate the sustainability of benefits in the longer term.

We also recognize that ReDIRECT is a complex intervention involving weight loss via TDR, dietitian and peer support: intervention participants had more human interactions over the 6 months, to which some benefits could be attributable. We did not collect blood samples from participants, so we cannot prove that weight loss reduced inflammation, a potential contributory mechanism for improving LC symptoms^24^. Indeed, given the lack of observed interaction in a crude post-hoc interaction analysis between weight loss and the primary outcome, other components of this complex intervention (for instance, the support provided by dietitians and peers) may be important mechanisms of symptom improvement.

The rate of noncompletion in the intervention group (24%) is on par with those reported for other dietary weight loss trials (22–25%) and lower than those reported in weight management services (for example 45% at 12 months in the service evaluation of the National Health Service Type 2 Diabetes Path to Remission Programme)^25–27^. Predictors of weight loss in trials have been shown to include percent weight loss in the first month, as well as age and previous weight loss attempts^25,26,28^. Baseline characteristics did not differ between completers and noncompleters in this study, except for levels of socioeconomic deprivation, with a greater proportion of people from IMD 1 and 2 categories for whom the primary outcome was not collected. Although it is possible that the higher number of people who did not complete the trial (at 6 months) in the intervention group could have introduced bias in the inference on the primary outcome, our multiple imputations using chained equations confirmed the findings. This will be explored further in a follow-up exploratory analysis of the trial outcomes.

While the remote/digital program offers many advantages to participants with fatigue and mobility issues, this service might present obstacles for people who lack internet access or digital skills who would not have volunteered for the study. However, 92% of UK adults use the internet at home or elsewhere (7% at risk of digital exclusion), and some participants received assistance and support from household members^29^. Future studies should consider ways to overcome digital and other forms of exclusion, including making patient-reported outcomes more inclusive^30^.

A strength of the trial remote design, ReDIRECT generated considerable interest across the United Kingdom and overrecruited within the allocated timeframe. Despite concerted efforts, men and ethnic minority groups remained underrepresented, limiting the generalizability of our findings. This potentially reflects the patient group living with LC (for example, fewer men have LC or engage with social media, patient groups or weight management)^31^. We note that the symptom outcome measures (for fatigue, pain, breathlessness, anxiety/depression and other) have not previously been validated in the context of LC, making it difficult to assess the clinical significance of improvements, but they have been widely used in other studies, including in people living with other energy-limiting conditions such as chronic fatigue syndrome or myalgic encephalopathy^32^. Over half of the participants chose fatigue as the symptom they would most like to improve. This is consistent with fatigue being reported as one of the most common symptoms in people living with LC (32–47%)^5,33^. With no specific statistics for populations living with both LC and overweight, it is impossible to establish whether our sample had a selection bias.

Patient and public involvement (PPI) was a particular strength, with involvement throughout the research. PPI collaborators informed the respective primary endpoint and enhanced the team’s understanding of LC symptoms, aiding the development of a weight management program that responded flexibly to the needs of people living with LC and overweight. We value the tremendous PPI input in this study and acknowledge the burden associated with involvement in the study. Our research team was particularly mindful of the impact of social media criticism on Long Covid Scotland as a third-sector organization and individual members of the broader team. Despite efforts to capture the impact of PPI electronically^34^, we note the resource-intensive nature of this task and the lack of suitable toolkits for dynamic studies.

This study is a randomized controlled trial of a weight management intervention for people living with both overweight and LC. Our findings are important since very few interventions have been shown to help people with LC. Our study design is also unique: treatments usually focus on a single symptom rather than addressing the symptom(s) that people find most troublesome or debilitating. ReDIRECT was, therefore, distinctive in adopting a respective primary outcome. This approach may have applications for research into other conditions. It should be noted that, although our analysis is powered for the respective primary outcome and the secondary outcomes of fatigue, breathlessness, pain and anxiety/depression (since each of these is measured for all participants at each time point), the study is not powered for subgroup analyses, and as such, these results should be viewed with caution.

Discussing weight management in the context of a secondary medical condition can be challenging for healthcare professionals, but there is a high acceptability of general practitioners raising the topic and offering help^35^. Not every person with LC is also living with overweight, and not all of those who are living with overweight would want to undertake weight loss as a strategy to manage a condition. For those prepared to undertake an evidence-based dietary weight management program, this study shows that such intervention can improve symptoms and quality of life. The symptoms that matter most to individuals improved significantly, with striking improvements in physical and mental fatigue, and people in the ReDIRECT intervention group experienced fewer missed hours of work. The implications for work productivity benefits, however, should be interpreted with caution given the relatively weak evidence for difference between groups at 6 months in terms of symptoms (*P* = 0.095), although the large difference in weight change between groups at 6 months may herald other benefits if weight loss is sustained longer. Further work is ongoing with observational data up to 12 months to determine more accurately the cost-effectiveness of this clinical management approach.

Our work has genuine translational potential for offering personalized dietary weight management solutions under existing services to people living with LC and excess weight, similar to how evidence-based weight management is offered for achieving T2 diabetes remission^12^. Important questions remain about the accessibility and acceptability of remotely delivered weight management interventions for people living with complex conditions across sociodemographic groups and the mediators of intervention effects, which remain to be fully explored to clarify the mechanism(s) of action of this complex intervention^36^.

In conclusion, these results represent robust randomized trial evidence that remotely delivered dietary weight management, which generates a mean of 10 kg intentional weight loss, is safe and effective in reducing the symptoms that matter most for people living with LC and overweight. Further studies are needed to determine longer-term outcomes and the mechanisms for observed benefits.

## Methods

### Study design

ReDIRECT was a randomized controlled trial with a wait-list design, with the control group granted delayed entry into the program after 6 months (ISRCTN registry 12595520). The study was delivered fully remotely, including recruitment across the United Kingdom, intervention delivery and self-reported data collection. Ethical approval was obtained from the South-East Scotland Research Ethics Committee 01 (REC reference number 21/SS/0077). The protocol is published elsewhere^38^.

### Participants

Eligible participants, aged ≥18 years, had a self-reported BMI >27 kg m^−2^ (>25 kg m^−2^ for South Asians), had a self-reported LC symptoms for >12 weeks before first recruitment contact and were not hospitalized, currently or for over 10 days during acute COVID-19 infection. A BMI of 25 kg m^−2^ in Asians is considered to have metabolic consequences similar to BMI 27 kg m^−2^ in Europeans^39^. A positive COVID-19 test or official LC diagnosis by a general practitioner was not required because the availability of COVID-19 tests was variable at the pandemic’s start and the emergence of LC as a new condition. Eligibility and inclusion criteria were guided by our PPI coapplicants and defined by the funder’s call requirements.

Exclusion criteria included hospitalizations >10 days or intensive care unit admissions related to COVID-19 (a prerequisite under the funding call); current use of insulin or antiobesity drugs; proven myocardial infarction within the past 6 months; severe mental illness (including severe depression and eating disorder); pregnancy or considering pregnancy; history of substance use; an active illness likely to cause a weight change; past (past 3 years) or planned bariatric surgery; established kidney disease (estimated glomerular filtration rate <50 ml min^−1^ 1.73 m^−2^), gallstones/pancreatitis; participation in another clinical research trial likely to affect diet or weight change; learning disabilities; and inability to understand English.

### Participant recruitment

Participants, male and female, were recruited across the United Kingdom via social media, online forums, LC networks, newspaper adverts and primary care records. Targeted efforts to recruit more men and ethnic minority groups included health promotion events at local football clubs, a local Men’s Shed initiative, places of worship and an interview on a local South Asian radio station.

People interested in participating were asked to contact the research team. They were emailed a participant information sheet and invited to a screening appointment by telephone or video, where inclusion and exclusion criteria were inventoried. To determine BMI, potential participants were asked to provide height and weight data at the remote screening by providing a recent weight measurement from the past 7 days or by weighing themselves during the call, and by measuring their height if unsure. Heights and weights not provided during the screening call were submitted after the screening via telephone, email or text message. All participants provided written informed consent, electronically.

### Randomization and masking

Participants were allocated to intervention or control groups using a mixed minimization and randomization approach to balance the groups with respect to the participant-selected dominant LC symptom (fatigue, breathlessness, pain, anxiety/depression or other), sex (male, female and other), age (<50 and 50+), ethnicity (white, South Asian and other) and IMD (postcode-based, deciles 1–5 and 6–10). Randomization took place after the completion of baseline assessments, using an online system developed and maintained by the Robertson Centre for Biostatistics (University of Glasgow, United Kingdom). Due to the nature of the diet intervention, participants, researchers and the dietitians who delivered the intervention were aware of group allocations. Statisticians analyzing the data remained blinded until the statistical analysis plan was completed and the primary analysis database was locked.

### Procedures

The intervention used in this study has been previously described in the protocol paper^38^.

Participants in the intervention group were provided with the Counterweight-Plus program, an evidence-based structured dietary weight management program (Counterweight)^40^. Weight loss was induced using a formula low-energy diet providing ~850 kcal per day as TDR for the first 8–12 weeks, followed by a stepped food reintroduction for 4–12 weeks and monthly visits for up to 1 year to support long-term weight loss maintenance. Participants who did not accept or could not tolerate the TDR were offered alternative dietary options, including MR, low carbohydrate and low fat. Swaps between dietary approaches were facilitated to maximize adherence and weight loss.

A registered dietitian delivered the program remotely to each participant with digital support via the Counterweight app (measurement logging, progress tracking, goal setting, reflective journal, recipes, activity videos and advice). Dietitians received standardized training in Counterweight-Plus and motivational interviewing and study-specific training from the lead dietitian and research associates undertaking the study. Support appointments, using video, telephone or text chat, included an orientation and planning call one week before starting the program and then at weeks 0 (start of TDR), 1 and 3. Further follow-up appointments were scheduled monthly, with timing/content tailored to individual circumstances. Program pauses (a planned break from the intervention for 1 month that could be extended in agreement with the dietitian) were offered to accommodate medical, life or personal events that impacted program participation. Weekly educational content was released via the Counterweight app that covered three principles: nutrition, physical activity and behavior change. In addition, topics covered included sleep, stress, emotions and mindfulness. Underpinning the whole intervention is the Counterweight Behavioural Toolkit, developed from the COM-B model (capability (C), opportunity (O), motivation (M) – behavior (B)) of behavior change, using the behavior change techniques taxonomy version 1 (BCTTv1), 45 behavior change techniques were incorporated in the intervention, such as goal setting, environmental restructuring, social support and relapse prevention^41,42^.

As in the DiRECT trial, all oral antihypertensive and antidiabetic drugs were discontinued upon commencing TDR to prevent postural hypotension or hypoglycemia during rapid weight loss, with standard protocols for drug reintroduction if indicated^12,43^. Information about adverse events was collected throughout the intervention via direct reports from dietitians to the research team and via information collection at each study visit by the research team.

### PPI

The team worked closely with Long Covid Scotland to involve people with lived experience of LC from application to dissemination. An initial online survey of Long Covid Scotland members gauged interest in a weight management intervention; two members of Long Covid Scotland have been part of the study team throughout, with a third sitting on the Trial Steering Committee. A separate PPI group (*n* = 6) also provided input (for example, intervention design, choice of respective primary outcome, tailoring of the weight management delivery in collaboration with the dietetic provider, recruitment, data collection, shaping dissemination activities; see the Guidance for Reporting Involvement of Patients and the Public (GRIPP2) short form checklist, Supplementary Table 5)^34^.

### Measurements

Trial outcomes were self-measured remotely to minimize participant burden and maximize retention. Web-based questionnaires collected self-reported baseline characteristics and sociodemographic data (including sex), self-measurements (using digital scales (A&D Medical Model UC-502) and automated blood pressure monitors (Kinetik Wellbeing Model – WBP1)), LC symptoms, healthcare resource use, productivity (employment), out-of-pocket costs, alternative treatments, health-related quality of life and psychological outcomes. Participants entered data directly into bespoke electronic Case Report Forms (eCRF), verified by research staff, or entered by research staff following communication via email, text or phone. All data captured in the eCRF are described in the protocol paper^38^.

Additional data were captured by Counterweight (screening/program start dates, withdrawal date and reason, program switched to and date, and number of sachets) and securely transferred to the biostatisticians.

### Outcomes

All outcome data were collected at baseline and 3, 6 and 12 months. The impact of the intervention on the primary outcome was evaluated at 6 months, reported herein.

The primary outcome was a continuous measure derived from the symptom score for the most important LC symptom selected by each participant at baseline (fatigue, breathlessness, pain, anxiety/depression or other). Symptom scores were assessed using validated questionnaires for the core symptoms of fatigue (Chalder fatigue scale), breathlessness (Medical Research Council dyspnea scale), pain (P4 pain rating scale) and anxiety and depression (hospital anxiety and depression scale, HADS)^44–47^. Other symptoms could be added via free text boxes and were scored using a 10-point VAS.

Symptom scores were standardized by subtracting the baseline mean score and dividing by the s.d., within the subgroup that selected each symptom as the one they would most like to see improve. For example, for all those who selected fatigue as their main symptom, we calculated the mean and s.d. of their baseline Chalder fatigue scale scores. For these people, the primary outcome at all time points was the Chalder fatigue scale, standardized using these baseline mean and s.d. values. The same process was followed for those who selected breathlessness as the symptom they would most like to see improve, using the Medical Research Council dyspnea scale scores, and similarly for those who selected pain (using the P4 pain rating scale) and anxiety/depression (using the HADS total score). For those who selected an ‘other’ symptom, we took the VAS score for the specific symptom identified at baseline for each participant. Note that participants could record multiple ‘other’ symptoms at each time point, but once a symptom had been recorded, participants were asked to rate that symptom at all subsequent visits so the primary outcome could be calculated at all time points by tracking the VAS scores for the specific symptom that was identified at baseline as the main symptom for each participant.

By definition, the primary outcome had a mean of zero at baseline and an s.d. very close to 1. People with positive scores at baseline for the primary outcome had symptom scores that were worse than average compared with people who selected the same main symptom. Improvements in the primary outcome corresponded with negative changes over time. Someone with a primary outcome of zero at baseline and −0.5 at follow-up could be considered to have improved by an amount equal to one-half of the s.d. as measured at baseline.

Secondary outcomes included the core LC symptoms (fatigue, breathlessness, pain and anxiety/depression), all other LC symptoms reported, self-measured weight, height, blood pressure, work productivity and activity impairment, healthcare resource use, medication (prescribed and over the counter) and food and drink costs for the previous week^48^. Health-related quality of life was estimated using the EuroQol-5 Dimensions-5 Levels questionnaire (EQ-5D-5L), where the scores were mapped to EQ-5D-3L utility values for the United Kingdom and applied to the time spent in the study to estimate QALYs^49^. Hours of work missed due to health problems 7 days before each assessment were reported and used to estimate productivity gains. Any adverse events reported to the study team were logged, and SAEs were assessed for expectedness and relatedness.

### Statistical analysis

The study was designed to detect a mean between-group difference of 0.5 s.d. units in the primary outcome, considering this as a moderate effect size due to the absence of prior information on the minimum clinically important difference for this composite outcome. For 90% power at 5% statistical significance, 86 participants per group with outcome data were calculated as necessary. Accounting for potential attrition, 200 people were planned to be randomized. Successful recruitment initiatives and a 12-week extension resulted in the randomization of 235 participants. There were more losses to follow-up in the intervention arm at 6 months, with the primary analysis comprising 97 intervention participants and 117 controls, providing 95.2% power to detect a 0.5-s.d. difference based on a two-sample *t*-test. Assuming some correlation between baseline and 6-month values for the primary outcome, using baseline-adjusted regression (analysis of covariance) will increase the power of the study further.

According to the intention-to-treat principle, analyses were done per a prespecified analysis plan.

Primary and secondary outcomes were analyzed using linear regression, with a binary predictor for the randomized group and adjustment for stratification variables (age (<50 years and ≥50 years), deprivation (deciles 1–5 and 6–10), sex, ethnicity (South Asian and other) and chosen symptom (fatigue, breathlessness, anxiety/depression, pain and other)) and the baseline value of the outcome. The exception was for those participants who reported at least one ‘other’ symptom at baseline: a linear mixed-effects model was fitted within each visit with a random effect of participant ID and fixed effects as described for the linear regression plus chosen symptom. All analyses report intervention effect estimates with 95% CIs and *P* values. No adjustments were made for multiple statistical comparisons.

The investigators examined the ‘other’ symptoms and recoded any better suited to the four main symptoms; for example, ‘fatigued’ was recoded as ‘fatigue’. A sensitivity primary s.d. score was calculated using these along with the rest of the participants’ original symptoms and analyzed in a linear regression as described above as a sensitivity analysis.

Missing data were not imputed in the main analysis, but multiple imputations using chained equations were applied as sensitivity analyses. Data were imputed separately within the intervention and control groups using the stratification variables and BMI, education, physical activity at baseline, employment change and EuroQol-5 Dimensions (EQ-5D) health utility. One-hundred imputations were carried out. A linear model for the primary s.d. scores, fatigue, breathlessness, anxiety/depression and EQ-5D was then performed on the imputed datasets, adjusting for stratification variables and baseline measurement, and these results were pooled. A linear mixed-effects model was performed on each imputed dataset for other symptoms, and the results were pooled.

For the primary outcome, subgroup analyses were carried out according to age tertile, IMD, BMI, sex, chosen symptom and fatigue selected as the chosen symptom. Regression models were extended to include interactions between the subgrouping variable and the intervention effect. Within-subgroup intervention effects and interaction *P* values are reported in a forest plot.

Inverse probability weighting was performed by using logistic regression to calculate each participant’s probability of follow-up and using the inverse of this probability as a weight in linear regressions for intervention effect adjusted as before.

The treatment effect on outcome given weight change at 3 or 6 months from baseline was analyzed using analysis of covariance with and without an interaction term in the model.

Statistical analyses were performed using SAS software, version 9.4 and R for Windows, version 4.1.2^50^.

### Participant withdrawal and loss to follow-up

Participants who withdrew from the weight management program (treatment) continued the study and provided data for the online questionnaire time points unless they specifically withdrew consent.

Nonresponders were contacted regularly by email, phone or text message up to 3 months after the scheduled questionnaire date. All nonresponders were considered on a case-by-case basis before they were declared as lost to follow-up. Participants categorized as lost to follow-up were contacted at least three times for subsequent questionnaires.

### Reporting summary

Further information on research design is available in the Nature Portfolio Reporting Summary linked to this article.

## Online content

Any methods, additional references, Nature Portfolio reporting summaries, source data, extended data, supplementary information, acknowledgements, peer review information; details of author contributions and competing interests; and statements of data and code availability are available at 10.1038/s41591-024-03384-x.

## Supplementary information

Supplementary Information Supplementary Tables 1–7: baseline characteristics, all outcomes at 3 months, all outcomes at 6 months, sensitivity analyses, GRIPP2 reporting checklist, Consolidated Standards of Reporting Trials (CONSORT) checklist and CONSORT extension for reporting of patient-reported outcomes (CONSORT-PRO) checklist.

Reporting Summary

## Data Availability

As per our study protocol, access to the raw data is restricted to the primary research team while the research is being conducted (until the end of the project, May 2024) and publication of the primary research papers. Upon publication of these papers, fully anonymized and minimized data (and data dictionaries) will be placed in a research data repository with access given to bona fide researchers on request to the corresponding authors and subject to appropriate data-sharing agreements. Proposals will be assessed on a monthly basis, with a response within 2 months of submission. The Statistical Analysis Plan is available via ISRCTN (10.1186/ISRCTN12595520), and ReDIRECT study eCRF questionnaire screenshots are available via figshare at 10.6084/m9.figshare.21270837 (ref. ^51^).
