# Supplementary material for: Remotely delivered weight management for people with long COVID and overweight: the randomized wait-list-controlled ReDIRECT trial
Source: Nat Med. 2025 Jan 8;31(1):258–66. doi: 10.1038/s41591-024-03384-x (PMC11750722; doi:10.1038/s41591-024-03384-x)
Supplement: Supplementary file 1 — Supplementary Tables 1–7: baseline characteristics, all outcomes at 3 months, all outcomes at 6 months, sensitivity analyses, GRIPP2 reporting checklist, Consolidated Standards of Reporting Trials (CONSORT) checklist and CONSORT extension for reporting of patient-reported outcomes (CONSORT-PRO) checklist. [file 41591_2024_3384_MOESM1_ESM.pdf]

nature medicine

Supplementary information https://doi.org/10.1038/s41591-024-03384-x

Remotely delivered weight management for people with long COVID and overweight: the randomized wait-list-controlled ReDIRECT trial

In the format provided by the authors and unedited

Table S1. Baseline characteristics for participants lost to follow-up at 6 months (time of primary analysis)

	Primary outcome not available (n=20)	Primary outcome available (n=97 intervention + 117 control)	p-value
**Sex, n (%)**			0.1986
Female	16 (80%)	182 (85%)	
Male	3 (15%)	31 (14.5%)	
Other	1 (5%)	1 (0.5%)	
**Ethnicity ǂ, n (%)**			1.0000
White	19 (95%)	192 (89.7%)	
South Asian	1 (5%)	9 (4.2%)	
Other Asian or Asian British	0	5 (2.3%)	
Black, African, Caribbean or Black British	0	2 (0.9%)	
Other or mixed ethnic group	0	6 (2.8%)	
**Median Age (years)**	45 (40, 51)	47 (40, 54)	0.4640
**IMD quintile, n (%)**			0.0055
1 (most deprived)	8 (40%)	23 (10.7%)	
2	5 (25%)	39 (18.2%)	
3	2 (10%)	43 (20.1%)	
4	1 (5%)	50 (23.4%)	
5 (least deprived)	4 (20%)	59 (27.6%)	
**Region, n (%)**			0.8313
England	12 (60%)	137 (64%)	
Scotland	7 (35%)	64 (29.9%)	
Wales	1 (5%)	10 (4.7%)	
Northern Ireland	0	3 (1.4%)	
**Median weight (kg)**	101.1 (90.5, 116.0)	97.7 (85.5, 113.8)	0.5675
**Median body mass index (BMI) (kg/m2)**	37.0 (33.8, 40.7)	35.1 (31.3, 40.4)	0.2977
BMI White (data unavailable: n=19, 95%; data available: n=192, 89.7%)	37.0 (33.8, 41.1)	35.1 (31.7, 40.4)	
BMI Non-white ǂ (data unavailable: n=1, 5%; data available: n=22, 10.3%)	29.4 (29.4, 29.4)	33.5 (29.5, 40.5)	
**Median Systolic blood pressure (mmHg)**	128 (118, 138)	130 (122. 139)	0.2607
**Median Diastolic blood pressure (mmHg)**	78 (71, 86)	80 (74, 86)	0.4732
**Hypertension, n (%)**	1 (5%)	31 (14.5%)	0.3252
**Known Type 2 diabetes mellitus, n (%)**	0	6 (2.8%)	1.0000
**Nominated long COVID symptom (primary outcome), n (%)**			0.5903
Pain	2 (10%)	26 (12.1%)	
Breathlessness	3 (15%)	34 (15.9%)	
Fatigue	11 (55%)	115 (53.7%)	
Anxiety/depression	1 (5%)	2 (0.9%)	
Other	3 (15%)	37 (17.3%)	
**Median time since 1st (reported) COVID infection (months)**	16 (7, 23)	18 (10, 24)	0.7246
**Median time since becoming aware of long COVID (months)**	14 (6, 21)	423 (223)15 (6, 21)	0.9039
**Taking medications§ n (%)**			0.3731
0	7 (35%)	36 (16.8%)	
1	1 (5%)	34 (15.9%)	
2	3 (15%)	22 (10.3%)	
3	2 (10%)	26 (12.1%)	
4	2 (10%)	25 (11.7%)	
5 or more	5 (25%)	71 (33.2%)	

Data are n (%) or median (IQR), unless otherwise specified. ǂ Ethnicities reported as non-white include South Asian, other Asians and Asian British. § Medication includes prescribed and over-the-counter. P-values are from Fisher's tests for categorical variables or Mann-Whitney tests for continuous variables. All p-values are two-tailed, and no adjustment is made for multiple comparisons.

Table S2. All outcomes at 3 months

Note: The primary statistical analysis of the ReDIRECT study was conducted at 6 months (Table S3).

	n (0M)	n (3M)	Mean (SD)			Intervention effect*	
			Baseline	3 months	Change	Estimate (95% CI)	p value
Primary outcome (main LC symptom)			-	-	-	-0.90 (-1.27, -0.53)	<0.0001
Intervention	116	101	0.0 (0.97)	-1.69 (1.91)	-1.66 (1.78)	-	-
Control	118	118	0.0 (1.02)	-0.79 (1.29)	-0.79 (1.12)	-	-
Weight			-	-	-	-8.47 (-9.87, -7.07)	<0.0001
Intervention	116	99	102.4 (21.5)	91.3 (19.8)	-9.8 (6.0)	-	-
Control	118	117	101.5 (19.4)	99.9 (19.4)	-1.3 (4.7)	-	-
BMI			-	-	-	-3.01 (-3.50, -2.52)	<0.0001
Intervention	116	99	36.8 (7.5)	32.9 (7.2)	-3.5 (2.0)	-	-
Control	118	117	36.8 (6.5)	36.3 (6.5)	-0.5 (1.7)	-	-
SBP			-	-	-	-6.74 (-9.87, -3.62)	<0.0001
Intervention	116	99	130.7 (14.5)	121.6 (12.5)	-9.2 (13.2)	-	-
Control	118	117	132.2 (13.3)	129.3 (13.7)	-3.0 (13.7)	-	-
DBP			-	-	-	-2.37 (-4.61, -0.14)	0.0378
Intervention	116	99	80.1 (9.1)	76.0 (10.0)	-4.4 (9.5)	-	-
Control	118	117	80.1 (8.8)	78.3 (9.0)	-1.8 (9.2)	-	-
Fatigue (total)			-	-	-	-5.89 (-7.73, -4.05)	<0.0001
Intervention	116	99	27.0 (4.4)	17.2 (9.3)	-9.8 (8.4)	-	-
Control	118	118	26.7 (5.0)	23.0 (6.1)	-3.8 (4.8)	-	-
Physical fatigue (subscale)			-	-	-	-3.94 (-5.20, -2.67)	<0.0001
Intervention	116	99	17.5 (3.0)	10.8 (6.1)	-6.7 (5.7)	-	-
Control	118	118	17.4 (3.1)	14.7 (4.1)	-2.7 (3.5)	-	-
Mental fatigue (subscale)			-	-	-	-1.92 (-2.65, -1.18)	<0.0001
Intervention	116	99	9.5 (2.0)	6.4 (3.6)	-3.1 (3.3)	-	-
Control	118	118	9.3 (2.4)	8.3 (2.6)	-1.1 (2.2)	-	-
Breathlessness			-	-	-	-0.23 (-0.43, -0.04)	0.0204
Intervention	116	100	1.8 (0.8)	1.4 (0.9)	-0.4 (0.8)	-	-
Control	118	118	1.9 (0.8)	1.7 (0.9)	-0.2 (0.8)	-	-
Pain			-	-	-	-2.32 (-4.18, -0.45)	0.0154
Intervention	116	99	19.7 (9.7)	14.9 (10.6)	-4.5 (7.5)	-	-
Control	118	118	19.4 (10.1)	17.3 (9.6)	-2.1 (7.2)	-	-
Hospital Anxiety and Depression Scale			-	-	-	-3.23 (-4.66, -1.79)	<0.0001
Intervention	116	99	19.7 (7.1)	15.9 (7.7)	-3.7 (6.6)	-	-
Control	118	118	19.7 (8.0)	19.3 (8.0)	-0.4 (4.8)	-	-
Anxiety (subscale)			-	-	-	-1.02 (-1.86, -0.19)	0.0167
Intervention	116	99	9.5 (4.4)	8.1 (4.4)	-1.3 (3.7)	-	-
Control	118	118	9.2 (4.8)	9.0 (4.9)	-0.2 (3.0)	-	-
Depression (subscale)			-	-	-	-2.21 (-3.03, -1.40)	<0.0001
Intervention	116	99	10.2 (3.7)	7.8 (4.2)	-2.5 (3.7)	-	-
Control	118	118	10.5 (4.0)	10.2 (4.1)	-0.2 (2.7)	-	-
Other LC symptoms **			-	-	-	-0.72 (-1.27, -0.17)	0.0113
Intervention	90	82	6.9 (2.2)	5.1 (2.9)	-1.8 (2.9)	-	-
Control	89	89	7.1 (2.2)	5.5 (2.6)	-1.5 (2.4)	-	-
Quality of life (EQ5D VAS)			-	-	-	6.62 (2.40, 10.85)	0.0023
Intervention	116	99	45.4 (17.2)	55.9 (21.4)	11.2 (17.6)	-	-
Control	118	118	46.2 (17.0)	49.9 (18.1)	3.8 (14.6)	-	-
Quality of life (EQ5D health utility score)			-	-	-	0.07 (0.02, 0.12)	0.0041
Intervention	116	99	0.48 (0.24)	0.55 (0.27)	0.09 (0.20)	-	-
Control	118	118	0.47 (0.27)	0.48 (0.27)	0.01 (0.17)	-	-

* Intervention effects are reported as the mean group differences based on linear regression adjusted for age, deprivation, sex, ethnicity, chosen symptom and the baseline value of the outcome. All p values are two-sided. No adjustment is made for multiple comparisons.** At baseline, n = 179 participants reported n = 816 other symptoms. Mean (SD) number of other symptoms per participant at baseline was 4 (4) in the intervention group and 3 (4) in the control group. Minimum and maximum values were (0, 25) and (0, 20) in the intervention and control groups. At 3 months, data were available for n = 788 symptoms across n = 171 participants.

0M: 0 months (baseline), 3M: 3 months (follow-up), BMI: body mass index, DBP: diastolic blood pressure, EQ-5D: EuroQol 5-dimension 5-level, LC: Long COVID, SBP: systolic blood pressure. Fatigue, Breathlessness, Pain, Anxiety/Depression, and quality of life were measured using the Chalder Fatigue Scale, Modified MRC Dyspnoea Scale, P4 Numeric Pain Rating Scale, Hospital Anxiety and Depression Scale, and EQ-5D-5L health utility score and visual analogue scale.

Table S3. All outcomes at 6 months

	n (0M)	n (6M)	Mean (SD)			Intervention effect*	
			Baseline	6 months	Change	Estimate (95% CI)	p value
Primary outcome (main LC symptom)			-	-	-	-0.34 (-0.67, -0.01)	0.0466
Intervention	116	97	0.0 (0.97)	-1.21 (1.69)	-1.16 (1.42)	-	-
Control	118	117	0.0 (1.02)	-0.82 (1.29)	-0.83 (1.14)	-	-
Weight			-	-	-	-9.79 (-11.5, -8.11)	<0.0001
Intervention	116	95	102.4 (21.5)	90.6 (20.0)	-10.3 (7.5)	-	-
Control	118	117	101.5 (19.4)	101.0 (19.4)	-0.7 (5.2)	-	-
BMI			-	-	-	-3.52 (-4.11, -2.94)	<0.0001
Intervention	116	95	36.8 (7.5)	32.5 (7.2)	-3.7 (2.6)	-	-
Control	118	117	36.8 (6.5)	36.6 (6.5)	-0.2 (1.8)	-	-
SBP			-	-	-	-4.16 (-7.42, -0.90)	0.0126
Intervention	116	95	130.7 (14.5)	124.0 (14.2)	-6.6 (14.1)	-	-
Control	118	117	132.2 (13.3)	129.0 (12.9)	-3.2 (12.9)	-	-
DBP			-	-	-	-2.73 (-4.95, -0.51)	0.0160
Intervention	116	95	80.1 (9.1)	75.6 (9.7)	-4.7 (9.0)	-	-
Control	118	117	80.1 (8.8)	78.2 (8.9)	-1.8 (9.1)	-	-
Fatigue (total)			-	-	-	-3.64 (-5.42, -1.86)	<0.0001
Intervention	116	97	27.0 (4.4)	19.4 (8.5)	-7.5 (7.7)	-	-
Control	118	117	26.7 (5.0)	23.1 (6.0)	-3.7 (5.5)	-	-
Physical fatigue (subscale)			-	-	-	-2.56 (-3.77, -1.35)	<0.0001
Intervention	116	97	17.5 (3.0)	12.1 (5.8)	-5.4 (5.4)	-	-
Control	118	117	17.4 (3.1)	14.7 (4.0)	-2.6 (3.6)	-	-
Mental fatigue (subscale)			-	-	-	-1.06 (-1.76, -0.36)	0.0032
Intervention	116	97	9.5 (2.0)	7.3 (3.1)	-2.2 (3.0)	-	-
Control	118	117	9.3 (2.4)	8.3 (2.6)	-1.1 (2.6)	-	-
Breathlessness			-	-	-	-0.27 (-0.48, -0.06)	0.0124
Intervention	116	97	1.8 (0.8)	1.4 (0.9)	-0.4 (0.9)	-	-
Control	118	117	1.9 (0.8)	1.8 (0.9)	-0.2 (0.8)	-	-
Pain			-	-	-	-1.41 (-3.32, 0.50)	0.1480
Intervention	116	97	19.7 (9.7)	16.3 (11.3)	-3.4 (7.5)	-	-
Control	118	117	19.4 (10.1)	17.5 (9.8)	-2.0 (7.0)	-	-
Hospital Anxiety and Depression Scale			-	-	-	-1.94 (-3.64, -0.25)	0.0249
Intervention	116	97	19.7 (7.1)	16.7 (9.2)	-2.9 (7.2)	-	-
Control	118	117	19.7 (8.0)	18.8 (8.6)	-0.9 (5.5)	-	-
Anxiety			-	-	-	-0.50 (-1.43, 0.43)	0.2947
Intervention	116	97	9.5 (4.4)	8.5 (5.0)	-0.7 (4.0)	-	-
Control	118	117	9.2 (4.8)	9.1 (4.8)	-0.2 (3.3)	-	-
Depression			-	-	-	-1.44 (-2.44, -0.43)	0.0052
Intervention	116	97	10.2 (3.7)	8.2 (5.0)	-2.2 (4.2)	-	-
Control	118	117	10.5 (4.0)	9.8 (4.6)	-0.7 (3.3)	-	-
Other LC symptoms **			-	-	-	-0.82 (-1.46, -0.19)	0.0113
Intervention	90	80	6.9 (2.2)	4.8 (2.9)	-2.0 (2.9)	-	-
Control	89	88	7.1 (2.2)	5.7 (2.8)	-1.4 (2.6)	-	-
Quality of life (EQ5D VAS)			-	-	-	7.55 (2.82, 12.29)	0.0019
Intervention	116	97	45.4 (17.2)	55.1 (22.5)	10.9 (18.2)	-	-
Control	118	117	46.2 (17.0)	48.4 (18.0)	2.3 (17.8)	-	-
Quality of life (EQ5D health utility score)			-	-	-	0.05 (-0.00, 0.11)	0.0725
Intervention	116	97	0.48 (0.24)	0.53 (0.29)	0.07 (0.23)	-	-
Control	118	117	0.47 (0.27)	0.49 (0.30)	0.01 (0.19)	-	-

* Intervention effects are reported as the mean group differences based on linear regression adjusted for age, deprivation, sex, ethnicity, chosen symptom and the baseline value of the outcome. All p values are two-sided. No adjustment is made for multiple comparisons.
** At baseline, n = 179 participants reported n = 816 other symptoms. Mean (SD) number of other symptoms per participant at baseline was 4 (4) in the intervention group and 3 (4) in the control group. Minimum and maximum values were (0, 25) and (0, 20) in the intervention and control groups. At 6 months, data were available for n = 764 symptoms across n = 168 participants.
0M: 0 months (baseline), 6M: 6 months (follow-up), BMI: body mass index, DBP: diastolic blood pressure, EQ-5D: EuroQol 5-dimension 5-level, LC: Long COVID, SBP: systolic blood pressure. Fatigue, Breathlessness, Pain, Anxiety/Depression, and quality of life were measured using the Chalder Fatigue Scale, Modified MRC Dyspnoea Scale, P4 Numeric Pain Rating Scale, Hospital Anxiety and Depression Scale, and EQ-5D-5L health utility score and visual analogue scale.

Table S4. Sensitivity analyses: Multiple Imputation (MI) and Inverse Probability of Follow-Up (IP)

Model	Main analysis Intervention Effect		MI Intervention Effect		IP Intervention Effect	
	Estimate (95% CI)	p-value	Estimate (95% CI)	p-value	Estimate (95% CI)	p-value
Primary outcome 6 months	-0.34 (-0.67, -0.01)	0.0466	-0.37 (-0.75, 0.01)	0.0568	-0.33 (-0.66, 0.01)	0.0557
Primary outcome 3 months	-0.90 (-1.27, -0.53)	<0.0001	-0.82 (-1.21, -0.43)	<0.0001	-0.94 (-1.31, -0.56)	<0.0001
Primary outcome sensitivity – “Other” symptom category recoded 6 months	-0.37 (-0.70, -0.03)	0.0342	-0.39 (-0.77, -0.01)	0.0451	-0.35 (-0.69, -0.01)	0.0413
Primary outcome sensitivity – “Other” symptom category recoded 3 months	-0.94 (-1.31, -0.56)	<0.0001	-0.85 (-1.24, -0.46)	<0.0001	-0.96 (-1.34, -0.58)	<0.0001
Weight 6 months	-9.80 (-11.5, -8.11)	<0.0001	-9.03 (-10.97, -7.09)	<0.0001	-9.7 (-11.4, -8.00)	<0.0001
Weight 3 months	-8.47 (-9.87, -7.07)	<0.0001	-8.04 (-9.45, -6.62)	<0.0001	-8.41 (-9.80, -7.01)	<0.0001
BMI 6 months	-3.52 (-4.11, -2.94)	<0.0001	-3.35 (-4.04, -2.66)	<0.0001	-3.49 (-4.09, -2.90)	<0.0001
BMI 3 months	-3.01 (-3.50, -2.52)	<0.0001	-2.86 (-3.37, -2.35)	<0.0001	-3 (-3.49, -2.51)	<0.0001
SBP 6 months	-4.16 (-7.42, -0.90)	0.0126	-4.01 (-7.50, -0.52)	0.0255	-4.11 (-7.35, -0.88)	0.0130
SBP 3 months	-6.74 (-9.87, -3.62)	<0.0001	-6.72 (-9.81, -3.62)	<0.0001	-6.51 (-9.60, -3.42)	<0.0001
DBP 6 months	-2.73 (-4.95, -0.51)	0.0160	-2.18 (-4.64, 0.28)	0.0844	-2.64 (-4.84, -0.43)	0.019.
DBP 3 months	-2.37 (-4.61, -0.14)	0.0378	-1.82 (-4.18, 0.53)	0.1309	-2.13 (-4.38, 0.12)	0.0628
Pain 6 months	-1.41 (-3.32, 0.50)	0.1480	-1.59 (-3.66, 0.49)	0.1354	-1.51 (-3.42, 0.40)	0.1211
Pain 3 months	-2.32 (-4.18, -0.45)	0.0154	-2.40 (-4.31, -0.49)	0.0148	-2.47 (-4.33, -0.62)	0.0092
Breathlessness 6 months	-0.27 (-0.48, -0.06)	0.0124	-0.26 (-0.47, -0.05)	0.0148	-0.27 (-0.48, -0.06)	0.0105
Breathlessness 3 months	-0.23 (-0.43, -0.04)	0.0204	-0.27 (-0.46, -0.08)	0.0068	-0.24 (-0.43, -0.04)	0.0163
Fatigue 6 months	-3.64 (-5.42, -1.86)	<0.0001	-4.20 (-6.09, -2.31)	<0.0001	-3.61 (-5.41, -1.81)	<0.0001
Fatigue 3 months	-5.89 (-7.73, -4.05)	<0.0001	-5.85 (-7.71, -4.00)	<0.0001	-6.11 (-7.96, -4.26)	0.0001
Fatigue (mental) 6 months	-1.06 (-1.76, -0.36)	0.0032	-1.21 (-1.98, -0.44)	0.0026	-1.05 (-1.76, -0.35)	0.0035
Fatigue (mental) 3 months	-1.92 (-2.65, -1.18)	<0.0001	-1.91 (-2.68, -1.14)	<0.0001	-2.05 (-2.80, -1.30)	<0.0001
Fatigue (physical) 6 months	-2.56 (-3.77, -1.35)	<0.0001	-3.00 (-4.40, -1.59)	<0.0001	-2.54 (-3.76, -1.31)	<0.0001
Fatigue (physical) 3 months	-3.94 (-5.20, -2.67)	<0.0001	-3.94(-5.27, -2.61)	<0.0001	-4.02 (-5.29, -2.76)	<0.0001
HADS 6 months	-1.94 (-3.64, -0.25)	0.02496	-2.03(-3.81, -0.25)	0.0270	-2.02 (-3.73, -0.30)	0.0215
HADS 3 months	-3.23 (-4.66, -1.79)	<0.0001	-3.16 (-4.59, -1.73)	<0.0001	-3.49 (-4.94, -2.04)	<0.0001
HADS anxiety 6 months	-0.50 (-1.43, 0.43)	0.2947	-0.382 (-1.38, 0.62)	0.4553	-0.53 (-1.47, 0.41)	0.2712
HADS anxiety 3 months	-1.02 (-1.86, -0.19)	0.0167	-0.97 (-1.84, -0.10)	0.0304	-1.14 (-1.98, -0.31)	0.0075
HADS depression 6 months	-1.44 (-2.44, -0.43)	0.0052	-1.61 (-2.72, -0.51)	0.0048	-1.48 (-2.50, -0.47)	0.0043
HADS depression 3 months	-2.21 (-3.03, -1.40)	<0.0001	-2.25 (-3.11, -1.39)	<0.0001	-2.35 (-3.18, -1.53)	<0.0001
Other 6 months	-0.82 (-1.46, -0.19)	0.0113	-0.94 (-1.62, -0.26)	0.0070	-0.83 (-1.46, -0.19)	0.0114
Other 3 months	-0.72 (-1.27, -0.17)	0.0113	-0.80 (-1.39, -0.20)	0.0089	-0.72 (-1.28, -0.17)	0.0112
EQ5D VAS 6 months	7.55 (2.82, 12.29)	0.0019	6.76 (1.47, 12.05)	0.0134	7.47 (2.70, 12.24)	0.0023
EQ5D VAS 3 months	6.62 (2.40, 10.85)	0.0022	6.79 (2.21, 11.37)	0.0042	6.76 (2.54, 10.97)	0.0018
EQ5D utility 6 months	0.05 (-0.00, 0.11)	0.0725	0.05 (-0.02, 0.11)	0.1518	0.05 (-0.01, 0.11)	0.0825
EQ5D utility 3 months	0.07 (0.02, 0.12)	0.0041	0.07 (0.02, 0.12)	0.0012	0.07 (0.03, 0.12)	0.0027

Missing data were imputed using Multiple Imputation by Chained Equations (MICE) with 100 imputations. Data were imputed separately within the intervention and control groups using the stratification variables and BMI, education, physical activity at baseline, employment change, and EQ5D health utility. A linear model for the personalised primary outcome was then performed on the imputed datasets adjusting for stratification variables and baseline measurement, and these results pooled. P values are two-tailed and no adjustment is made for multiple comparisons.

Using the inverse probability of follow-up determined using logistic regression, primary and secondary outcomes were weighted. The logistic regression model used randomised treatment, chosen symptom, age (above and below 50), sex, IMD (above and below 5th decile), ethnicity, BMI, and baseline physical activity as covariates. Linear models were then constructed, weighted using these probabilities. P-values are two-tailed. No adjustment is made for multiple comparisons

Table S5. GRIPP2 Reporting Checklist – Short Form

Staniszewska S, Brett J, Simera I, Seers K, Mockford C, Goodlad S et al. GRIPP2 reporting checklists: tools to improve reporting of patient and public involvement in research. BMJ 2017; 358 :j3453 doi:10.1136/bmj.j3453

Section and topic	Item	Reported on page number
1. Aim	Report the aim of PPI in the study	10
2. Methods	Provide a clear description of the methods used for PPI in the study	10
3. Study results	Outcomes: Report the results of PPI in the study, including both positive and negative outcomes	10
4. Discussion and conclusion	Outcomes: Comment on the extent to which PPI influenced the study overall. Describe positive and negative effects	6
5. Reflections/critical perspective	Comment critically on the PPI in the study, reflecting on the things that went well and those that did not, so others can learn from this experience	6

Table S6. CONSORT 2010 Checklist - Remotely-delivered weight management for people living with long COVID and overweight (ReDIRECT): a wait-list controlled randomised trial

Section/Topic	Item No	Checklist item	Reported on page No
**Title and abstract**			
	1a	Identification as a randomised trial in the title	1
	1b	Structured summary of trial design, methods, results, and conclusions (for specific guidance see CONSORT for abstracts)	1
**Introduction**			
Background and objectives	2a	Scientific background and explanation of rationale	1-2
	2b	Specific objectives or hypotheses	2
**Methods**			
Trial design	3a	Description of trial design (such as parallel, factorial) including allocation ratio	1, 10
	3b	Important changes to methods after trial commencement (such as eligibility criteria), with reasons	N/A
Participants	4a	Eligibility criteria for participants	10
	4b	Settings and locations where the data were collected	10-11
Interventions	5	The interventions for each group with sufficient details to allow replication, including how and when they were actually administered	10
Outcomes	6a	Completely defined pre-specified primary and secondary outcome measures, including how and when they were assessed	10-11
	6b	Any changes to trial outcomes after the trial commenced, with reasons	N/A
Sample size	7a	How sample size was determined	11
	7b	When applicable, explanation of any interim analyses and stopping guidelines	N/A
Randomisation:			
Sequence generation	8a	Method used to generate the random allocation sequence	10
	8b	Type of randomisation; details of any restriction (such as blocking and block size)	10
Allocation concealment mechanism	9	Mechanism used to implement the random allocation sequence (such as sequentially numbered containers), describing any steps taken to conceal the sequence until interventions were assigned	10
Implementation	10	Who generated the random allocation sequence, who enrolled participants, and who assigned participants to interventions	10
Blinding	11a	If done, who was blinded after assignment to interventions (for example, participants, care providers, those assessing outcomes) and how	10
	11b	If relevant, description of the similarity of interventions	N/A
Statistical methods	12a	Statistical methods used to compare groups for primary and secondary outcomes	11-12
	12b	Methods for additional analyses, such as subgroup analyses and adjusted analyses	11-12
**Results**			
Participant flow (a diagram is strongly recommended)	13a	For each group, the numbers of participants who were randomly assigned, received intended treatment, and were analysed for the primary outcome	2-3
	13b	For each group, losses and exclusions after randomisation, together with reasons	2-3
Recruitment	14a	Dates defining the periods of recruitment and follow-up	2
	14b	Why the trial ended or was stopped	N/A
Baseline data	15	A table showing baseline demographic and clinical characteristics for each group	3
Numbers analysed	16	For each group, number of participants (denominator) included in each analysis and whether the analysis was by original assigned groups	2-3, Suppl S1-4
Outcomes and estimation	17a	For each primary and secondary outcome, results for each group, and the estimated effect size and its precision (such as 95% confidence interval)	3-4, Suppl S2-3
	17b	For binary outcomes, presentation of both absolute and relative effect sizes is recommended	N/A
Ancillary analyses	18	Results of any other analyses performed, including subgroup analyses and adjusted analyses, distinguishing pre-specified from exploratory	5, Suppl S2-4
Harms	19	All important harms or unintended effects in each group (for specific guidance see CONSORT for harms)	4, 6
**Discussion**			
Limitations	20	Trial limitations, addressing sources of potential bias, imprecision, and, if relevant, multiplicity of analyses	6-7
Generalisability	21	Generalisability (external validity, applicability) of the trial findings	6-7
Interpretation	22	Interpretation consistent with results, balancing benefits and harms, and considering other relevant evidence	5-7-
**Other information**			
Registration	23	Registration number and name of trial registry	1, 10
Protocol	24	Where the full trial protocol can be accessed, if available	10
Funding	25	Sources of funding and other support (such as supply of drugs), role of funders	12

Table S7. CONSORT PRO 2013 Checklist - Remotely-delivered weight management for people living with long COVID and overweight (ReDIRECT): a wait-list controlled randomised trial

Section/Topic	Item No	Checklist item	Reported on page No
**Title and abstract**			
	P1b	The PRO should be identified in the abstract as a primary or secondary outcome.	1
**Introduction**			
Background and objectives	2a	Scientific background and explanation of rationale of PRO assessment should be included	2
	P2b	The PRO hypothesis should be stated, and relevant domains identified, if applicable.	2
**Methods**			
Participants	4a	PRO-specific criteria are required only if PROs were used for eligibility or stratification.	10
Outcomes	P6a	Evidence of PRO instrument validity and reliability should be provided or cited if available including the person completing the PRO and methods of data collection (paper, telephone, electronic).	10-11
Sample size	7a	Sample size determination is required only if PRO is a primary study outcome.	11
**Randomisation**			
Statistical methods	P12a	Statistical approaches for dealing with missing data are explicitly stated.	11
**Results**			
Participant flow (a diagram is strongly recommended)	13a	The number of PRO outcome data at baseline and at subsequent time points should be transparent.	Figure 2 (primary outcome) and Supplementary Tables S2 and S3 for all outcomes
Baseline data	15	PRO data in the table showing baseline demographic and clinical characteristics for each group should be included.	Table 1
Numbers analysed	16	For each group, the number of participants (denominator) included in each analysis and whether the analysis was by original assigned groups) is required for PRO results.	2, 11, Supplementary Tables S2 and S3
Outcomes and estimation	17a	The estimated effect size and its precision such as 95% confidence interval should be presented for multidimensional PROs from each domain and time point.	3-4
Ancillary analyses	18	Results of any other PRO analyses performed, including subgroup analyses and adjusted analyses, distinguishing pre-specified from exploratory should be presented, where relevant.	5
**Discussion**			
Limitations	P20/21	PRO-specific limitations and implications for generalizability and clinical practice should be presented.	5-7
Interpretation	22	PRO data should be interpreted in relation to clinical outcomes including survival data, where relevant	6-7
