# Supplementary material for: Remotely delivered weight management for people with long COVID and overweight: the randomized wait-list-controlled ReDIRECT trial
Source: Nat Med. 2025 Jan 8;31(1):258–66. doi: 10.1038/s41591-024-03384-x (PMC11750722; doi:10.1038/s41591-024-03384-x)
Supplement: Supplementary file 2 — Reporting Summary [file 41591_2024_3384_MOESM2_ESM.pdf]

nature portfolio

Corresponding author(s): Emilie Combet and David Blane

Last updated by author(s): Oct 4, 2024

Reporting Summary

Nature Portfolio wishes to improve the reproducibility of the work that we publish. This form provides structure for consistency and transparency in reporting. For further information on Nature Portfolio policies, see our Editorial Policies and the Editorial Policy Checklist.

Statistics

For all statistical analyses, confirm that the following items are present in the figure legend, table legend, main text, or Methods section.

n/a	Confirmed	
☐	☒	The exact sample size (*n*) for each experimental group/condition, given as a discrete number and unit of measurement
☒	☐	A statement on whether measurements were taken from distinct samples or whether the same sample was measured repeatedly
☐	☒	The statistical test(s) used AND whether they are one- or two-sided<br>*Only common tests should be described solely by name; describe more complex techniques in the Methods section.*
☐	☒	A description of all covariates tested
☐	☒	A description of any assumptions or corrections, such as tests of normality and adjustment for multiple comparisons
☐	☒	A full description of the statistical parameters including central tendency (e.g. means) or other basic estimates (e.g. regression coefficient) AND variation (e.g. standard deviation) or associated estimates of uncertainty (e.g. confidence intervals)
☐	☒	For null hypothesis testing, the test statistic (e.g. *F*, *t*, *r*) with confidence intervals, effect sizes, degrees of freedom and *P* value noted<br>*Give P values as exact values whenever suitable.*
☒	☐	For Bayesian analysis, information on the choice of priors and Markov chain Monte Carlo settings
☐	☒	For hierarchical and complex designs, identification of the appropriate level for tests and full reporting of outcomes
☐	☒	Estimates of effect sizes (e.g. Cohen's *d*, Pearson's *r*), indicating how they were calculated

Our web collection on statistics for biologists contains articles on many of the points above.

Software and code

Policy information about availability of computer code

Data collection	Participants entered data directly into bespoke electronic Case Report Forms (eCRF) - ReDIRECT study eCRF questionnaires screenshots are available via Figshare (https://doi.org/10.6084/m9.figshare.21270837). Data linked to dietetic visits were entered in Excel spreadsheets.
Data analysis	Statistical analyses were performed using SAS software, version 9.4 and R for Windows, version 4.1.2

For manuscripts utilizing custom algorithms or software that are central to the research but not yet described in published literature, software must be made available to editors and reviewers. We strongly encourage code deposition in a community repository (e.g. GitHub). See the Nature Portfolio guidelines for submitting code & software for further information.

Data

Policy information about availability of data

All manuscripts must include a data availability statement. This statement should provide the following information, where applicable:

- Accession codes, unique identifiers, or web links for publicly available datasets
- A description of any restrictions on data availability
- For clinical datasets or third party data, please ensure that the statement adheres to our policy

As per our study protocol, access to the raw data is restricted to the primary research team whilst the research is being conducted (until the end of the project, May 2024) and publication of the primary research papers. Upon publication of these papers, fully anonymised and minimised data (and data dictionaries) will be placed in a research data repository with access given to bona fide researchers on request to the corresponding authors and subject to appropriate data-sharing

agreements. Proposals will be assessed on a monthly basis, with a response within 2 months of submission. The Statistical Analysis Plan is made available as Supplementary data and ReDIRECT study eCRF questionnaires screenshots are available via Figshare (https://doi.org/10.6084/m9.figshare.21270837).

Research involving human participants, their data, or biological material

Policy information about studies with human participants or human data. See also policy information about sex, gender (identity/presentation), and sexual orientation and race, ethnicity and racism.

Reporting on sex and gender	The study includes male and female participants, of which 85% were female. Sex is provided in the descriptive analysis and used for the subgroup analysis.
Reporting on race, ethnicity, or other socially relevant groupings	The study population included multiple ethnicities, and was mostly white (90%), ith people living in areas across quintiles of deprivations. Ethnicity and levels of socioeconomic deprivations are reported in the descriptive statistics and used in subgroup analyses.
Population characteristics	Participants were people living with Long COVID and overweight/obesity, living across the UK. Median age was 47 (min 19, max 81). Population characteristics are described in Tables 1 and S1 and in text.
Recruitment	Participants were recruited across the UK via social media, online forums, Long COVID networks, newspaper adverts, and primary care records. Targeted efforts to recruit more men and ethnic minority groups included health promotion events at local football clubs, a local Men's Shed initiative, places of worship, and an interview on a local South Asian radio station.<br><br>this was a fully remote intervention using digital technology - While the remote/digital programme offers many advantages to participants with fatigue and mobility issues, this service might present obstacles for people who lack internet access or digital skills who would not have volunteered for the study.
Ethics oversight	Ethical approval was obtained from the South-East Scotland Research Ethics Committee 01 (REC reference number: 21/SS/0077).

Note that full information on the approval of the study protocol must also be provided in the manuscript.

Field-specific reporting

Please select the one below that is the best fit for your research. If you are not sure, read the appropriate sections before making your selection.

☒ Life sciences ☐ Behavioural & social sciences ☐ Ecological, evolutionary & environmental sciences

For a reference copy of the document with all sections, see nature.com/documents/nr-reporting-summary-flat.pdf

Life sciences study design

All studies must disclose on these points even when the disclosure is negative.

Sample size	The study was designed to detect a mean between-group difference of 0.5 standard deviation (SD) units in the primary outcome, considering this as a moderate effect size due to the absence of prior information on the minimum clinically important difference for this composite outcome. For 90% power at 5% statistical significance, 86 participants per group with outcome data were calculated as necessary. Accounting for potential attrition, 200 people were planned to be randomised. Successful recruitment initiatives and a 12-week extension resulted in the randomisation of 235 participants.
Data exclusions	No data were excluded from the analysis
Replication	this is a randomised control trial, therefore replication is not applicable
Randomization	Participants were allocated to intervention or control groups using a mixed minimisation and randomisation approach to balance the groups with respect to the participant-selected dominant LC symptom (fatigue, breathlessness, pain, anxiety/depression, or other), sex (male, female, other), age (<50, 50+), ethnicity (White, South Asian, other), and index of multiple deprivation (postcode-based, deciles 1-5, 6-10). Randomisation took place after the completion of baseline assessments, using an online system developed and maintained by the Robertson Centre for Biostatistics (University of Glasgow, UK).
Blinding	Statisticians analysing the data remained blinded until the statistical analysis plan was completed and the primary analysis database locked.

Reporting for specific materials, systems and methods

We require information from authors about some types of materials, experimental systems and methods used in many studies. Here, indicate whether each material, system or method listed is relevant to your study. If you are not sure if a list item applies to your research, read the appropriate section before selecting a response.

Materials & experimental systems

n/a	Involved in the study
☒	☐ Antibodies
☒	☐ Eukaryotic cell lines
☒	☐ Palaeontology and archaeology
☒	☐ Animals and other organisms
☐	☒ Clinical data
☒	☐ Dual use research of concern
☒	☐ Plants

Methods

n/a	Involved in the study
☒	☐ ChIP-seq
☒	☐ Flow cytometry
☒	☐ MRI-based neuroimaging

Clinical data

Policy information about clinical studies

All manuscripts should comply with the ICMJE guidelines for publication of clinical research and a completed CONSORT checklist must be included with all submissions.

Clinical trial registration	ISRCTN registry 12595520
Study protocol	Haag L, et al. NIHR open research 2022; 2: 57. https://doi.org/10.3310/nihropenres.13315.2
Data collection	Participants were recruited and consented between 23 December 2021 and 04 July 2022. Data was collected remotely, from participants across the UK, using electronic case report forms.
Outcomes	The primary outcome was a continuous measure derived from the symptom score for the most important LC symptom selected by each participant at baseline (fatigue, breathlessness, pain, anxiety/depression, or other). Symptom scores were assessed using validated questionnaires for the core symptoms of fatigue (Chalder Fatigue Scale), breathlessness (MRC Dyspnoea Scale), pain (P4 Pain Rating Scale), anxiety and depression (Hospital Anxiety and Depression Scale). Other symptoms could be added via free text boxes and were scored using a 10-point visual analogue scale (VAS).<br><br>Secondary outcomes included the core LC symptoms (fatigue, breathlessness, pain, anxiety/depression), all "other" LC symptoms reported, self-measured weight, height, blood pressure, Work Productivity and Activity Impairment, healthcare resource use, medication (prescribed and over-the-counter) and food/drink costs for the previous week.

Plants

Seed stocks	N/A
Novel plant genotypes	N/A
Authentication	N/A
